# Risk Factors for Depressive Symptoms in Korean Adult Stroke Survivors: The Korea National Health and Nutrition Examination Survey IV–VII (2007–2018)

**DOI:** 10.3390/ijerph18158178

**Published:** 2021-08-02

**Authors:** Min-Woo Hong, Jong-Hwa Lee, Kyeong-Woo Lee, Sang-Beom Kim, Min-Gu Kang

**Affiliations:** 1 Department of Physical Medicine and Rehabilitation, Dong-A University College of Medicine, Busan 49201, Korea; kor17105@gmail.com (M.-W.H.); jhlee08@dau.ac.kr (J.-H.L.); kwlee65@hanmail.net (K.-W.L.); sbkim@dau.ac.kr (S.-B.K.); 2 Regional Cardiocerebrovascular Center, Dong-A University Hospital, Busan 49201, Korea

**Keywords:** stroke, depression, suicide, psychological distress

## Abstract

Depressive symptoms are common in stroke survivors, and they are associated with poor outcomes. Therefore, this study aimed to investigate the depressive symptoms in stroke survivors and the risk factors for depressive symptoms in stroke survivors. We included 33,991 participants who were 19 years or older and had completed a questionnaire about the history of stroke from the Korea National Health and Nutrition Examination Survey (KNHANES) IV–VII (from 2007 to 2018). The mean Patient Health Questionnaire-9 score and the prevalence of major depression, depressive symptoms, antidepressant treatment, suicidal ideation, and suicide attempts were significantly higher in stroke survivors than in non-stroke participants (4.4 vs. 2.6, 16.2% vs. 5.3%, 24.7% vs. 9.3%, 3.8% vs. 1.4%, 21.7% vs. 4.8%, and 2.5% vs. 0.6%, respectively, all *p* < 0.001). Complex sample multivariate logistic regression analysis revealed that the female sex, unemployment, a low education level, a low family income, and activity limitations were independent risk factors for depressive symptoms in stroke survivors. Activity limitations showed the highest odds ratio among the independent factors, and its causes were further analyzed. The most common causes of activity limitations were stroke sequelae and musculoskeletal problems. To reduce depressive symptoms in stroke survivors, attention needs to be paid to minimizing stroke sequelae and musculoskeletal problems along with regular screening for depressive symptoms.

## 1. Introduction

Stroke survivors have an increased incidence of neuropsychiatric disorders, such as depression and generalized anxiety disorder [1]. Depression is one of the most common neuropsychiatric disturbances following a stroke, and approximately one-third of stroke survivors suffer from depression [2,3,4]. Furthermore, stroke survivors’ depressive symptoms are negatively associated with survival, functional outcome, and quality of life [5,6]. Therefore, identifying stroke survivors who are susceptible to depressive symptoms is important for a better outcome.

Although many factors have been suggested as risk factors for depression in stroke patients, it is still unclear which factors are associated with depression [7,8,9,10]. Conflicting results have been reported on the relationship between depression and stroke characteristics, such as lesion location and lesion size [11,12]. A recent meta-analysis study reported that lesion location might be a significant risk factor, but not lesion laterality or type of stroke. In addition to stroke characteristics, demographics and socioeconomic characteristics may be significant risk factors for depression in stroke survivors [13]. Age, gender, education level, family income, social support, and functional impairment are commonly discussed risk factors. However, consistent results have not always been reported for these factors. Small sample sizes or differences in ethnic or cultural characteristics may have contributed to the inconsistent findings. Therefore, this study aimed to identify the risk factors for depressive symptoms in stroke survivors by using the Korea National Health and Nutrition Examination Survey (KNHANES).

## 2. Materials and Methods

### 2.1. Study Population

We analyzed data from the KNHANES IV (2007–2009), V (2010–2012), VI (2013–2015), and VII (2016–2018). The KNHANES is a nationwide, population-based, cross-sectional study that has been conducted periodically since 1998 by the Korea Centers for Disease Control and Prevention to assess the health and nutritional status of the Korean civilian and non-institutionalized population. This cross-sectional survey consists of a physical examination and a health interview, and it uses clustered, multistage, stratified, and probability sampling to represent the non-institutionalized civilian Korean population [14].

All participants provided written informed consent to participate in the KNAHNES. The study was approved by the Institutional Review Board of the Korea Centers for Disease Control and Prevention. The Institutional Review Board of Dong-A University Hospital determined that this study was exempt from requiring their approval (DAUHIRB-EXP-21-033).

### 2.2. Study Variables

The demographic variables included age, sex, employment status, education level, marital status, and family income. Clinical characteristics were collected by self-reported questionnaires, physical examinations, and laboratory analyses.

The questionnaire about the participants’ mental health included the EuroQol 5-Dimension 3-Level (EQ-5D-3L), the Patient Health Questionnaire-9 (PHQ-9), and the presence of antidepressant treatment, suicidal ideation, and attempt. The EQ-5D-3L is one of the most widely used instruments to measure health-related quality of life [15]. It comprises five dimensions: mobility, self-care, usual activities, pain/discomfort, and anxiety/depression. The PHQ-9 is a self-reported questionnaire that evaluates the presence and severity of depression [16]. It contains nine items, each of which is scored on a scale of 0–3, resulting in a total score ranging from 0 to 27. Higher scores indicate more severe depressive symptoms. The participants were asked the following questions about suicidal ideation and attempt: “During the past year, did you ever seriously consider attempting suicide?” and “During the past year, did you ever attempt suicide?”

### 2.3. Definition of Stroke Survivors and Depressive Symptoms

Participants who answered “Yes” to the question of “Have you ever been diagnosed with stroke by a doctor?” were defined as stroke survivors in this study.

The presence of depressive symptoms was determined using EQ-5D-3L’s anxiety/depression component. Responses were captured as (1) not anxious or depressed, (2) moderately anxious or depressed, and (3) extremely anxious or depressed. A score of 2 to 3 was classified as the presence of depressive symptoms.

### 2.4. Statistical Analysis

Participants were divided into two groups according to a history of stroke. Categorical variables were compared using the chi-square test, and continuous variables were compared using the independent t-test. The complex sample generalized linear model and complex sample Rao–Scott adjusted chi-square test were used to compare the mean PHQ-9 scores and the prevalence of major depression, depressive symptoms, suicidal ideation, and suicide attempts between stroke survivors and non-stroke participants. Complex sample univariate logistic regression analysis was used to investigate factors associated with depressive symptoms in stroke survivors by estimating the odds ratios (ORs) and 95% confidence intervals (CIs). Then, complex sample multivariate logistic regression analysis was performed to determine the risk factors. The statistical analysis was conducted using the complex sample analysis module of SPSS 20.0 (IBM Corp., Armonk, NY, USA).

## 3. Results

**Figure 1:**
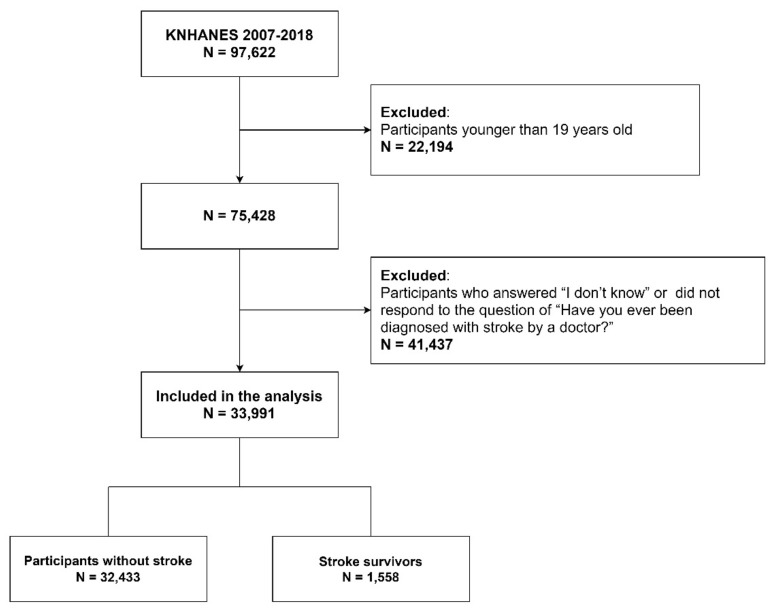
Flow diagram of the study participants.

Figure 1 shows the flow diagram of participants in the study. A total of 33,991 adults aged 19 years or older who answered “Yes” or “No” to the question of “Have you ever been diagnosed with stroke by a doctor?”.

Table 1 shows the participants’ demographic and clinical characteristics. The stroke survivors were significantly older than non-stroke participants (*p* < 0.001). The proportion of participants who were male, had a low education level, were unemployed, and had a low family income was higher in stroke survivors (all *p* < 0.001). The comorbidities, including hypertension, diabetes mellitus, and hyperlipidemia, were more prevalent in stroke survivors (all *p* < 0.001). Activity limitations and depressive symptoms were also more prevalent in stroke survivors (all *p* < 0.001).

### 3.1. Comparison of Mental Health

Table 2 presents the comparison of the mental health between the stroke survivors and non-stroke participants. The mean PHQ-9 score was higher in the stroke survivors (4.4 vs. 2.6, *p* < 0.001). The prevalence of major depression was higher in the stroke survivors (16.2% vs. 5.3%). Major depression was defined as a PHQ-9 score of 10 or above [17,18]. The prevalence of depressive symptoms, antidepressant treatment, suicidal ideation, and suicide attempts were greater in the stroke survivors than in non-stroke participants (24.7% vs. 9.3%, 3.8% vs. 1.4%, 21.7% vs. 4.8%, and 2.5% vs. 0.6%, respectively, all *p* < 0.001).

### 3.2. Risk Factors for Depressive Symptoms in Stroke Survivors

We performed complex sample univariate and multivariate logistic regression analyses to investigate the risk factors for depressive symptoms in stroke survivors (Table 3). In the univariate analysis, older age, the female sex, unemployment, a low education level, a low family income, and activity limitations were associated with depressive symptoms. The multivariate analysis showed that the female sex, unemployment, low education level, low family income, and activity limitations remained significant risk factors.

### 3.3. Causes of Activity Limitations

Activity limitations were identified as a significant risk factor for depressive symptoms with the highest odds ratio, and we further analyzed their causes (Table 4). The most common causes of activity limitations reported by the participants were stroke sequelae (53.9%) and musculoskeletal problems (41.0%), followed by visual problems (9.3%), hypertension (8.5%), and diabetes mellitus (7.2%).

## 4. Discussion

In this study, the mean PHQ-9 score and the prevalence of major depression, depressive symptoms, suicidal ideation, and suicide attempts were significantly higher in stroke survivors than in non-stroke participants. Complex sample multivariate logistic regression analysis revealed that the female sex, unemployment, a low education level, a low family income, and activity limitations were independent risk factors for depressive symptoms in stroke survivors. Activity limitations were identified as a significant risk factor with the highest odds ratio, and further analysis revealed that the most common causes of activity limitations were stroke sequelae and musculoskeletal problems. 

Being female was a significant risk factor for depressive symptoms in stroke survivors in the present study, which is in line with previous studies [8,19,20]. Depression is more common in women than in men in the general population, and pre-stroke depression is an important risk factor for post-stroke depression [21,22]. Therefore, the depressive symptoms that were more common in women before the stroke may have continued or worsened after the stroke. Moreover, sex hormones regulate neurotransmitter systems that affect sensitivity to the external environment, and differences in these hormones can lead to differences in adaptation patterns to environmental changes, which can create gender differences in depressive symptoms after stroke [21].

Aström et al. reported that living alone was a risk factor for post-stroke depression [23], and a meta-analysis revealed that social support was a protective factor, whereas the stroke severity and the level of handicap were risk factors for post-stroke depression [8]. Unemployment and activity limitations reflect the stroke severity and level of handicap and can lead to social isolation. This may explain why unemployment and activity limitations were identified as significant risk factors in the present study.

Socioeconomic status, including education and family income, was associated with depressive symptoms in this study. Higher education may lead to higher income and better access to health care service, healthy food, and a safe living environment [24,25]. High socioeconomic status can lead to enriched social networks that provide greater social support, protecting against depressive symptoms [26]. In addition, people with high socioeconomic status may have a greater sense of agency and self-esteem [27]. In contrast, low education may lead to more severe stroke, which in turn increases depressive symptoms [28]. Low education is also associated with white matter hyperintensities, which is known to be associated with depression [29]. 

Activity limitations were significantly associated with depressive symptoms in stroke survivors in the present study. Likewise, previous studies have reported significant associations between physical activity and depression. One possible mechanism underlying this relationship can be explained by self-efficacy theory [30], which proposes that continuing to engage in challenging activities can enhance an individual’s self-efficacy. For stroke survivors, where exercise can be difficult, regular physical activity can improve their self-confidence and mental health [31]. Another possible mechanism is the endorphin hypothesis [32]. Endorphins enable us to withstand pain and stress. Feelings of euphoria and analgesia have been reported after intense training, which are considered to be due to the effects of the secretion of endorphins [33]. The monoamine hypothesis, which is based on the fact that monoamine levels are low in depressed patients, is also a possible explanation [34]. Patients with depression have a high density of monoamine oxidase A, which non-specifically metabolizes monoamines [35]. Medications that inhibit the reuptake of monoamines, including serotonin and noradrenaline, improve depressive symptoms by increasing the availability of monoamines in the brain [36]. Similar to antidepressants, exercise improves depressive symptoms by increasing the brain’s levels of serotonin and adrenaline [37,38]. 

Benito-Montagut et al. and Cattan et al. defined social isolation as the absence of meaningful contact with individuals or communities [39,40]. Many elderly people are not sufficiently physically active to engage in activities that facilitate social interaction [41]. Stroke survivors who are not only elderly but also have activity limitations are likely to have difficulty with social interaction. This might aggravate their social isolation, which is reported to be a risk factor for depressive symptoms in stroke survivors [42].

Due to the abovementioned mechanisms and explanations, activity limitations can lead to depressive symptoms in stroke survivors. As such, it is necessary to pay attention to stroke sequelae and musculoskeletal problems, which are the main causes of activity limitations. Therefore, in order to reduce the depressive symptoms in stroke survivors, clinicians should consider screening for mental health and musculoskeletal problems as well. Both acute management to minimize stroke sequelae and chronic management to identify and manage musculoskeletal problems are essential to diminish depressive symptoms in stroke survivors.

This study has several limitations. First, stroke history was only confirmed by the participants’ questionnaire, not by medical records. Therefore, stroke characteristics such as severity, lesion location, and type of stroke could not be analyzed. Second, this study investigated the risk factors for depressive symptoms, not for major depression. PHQ-9 was investigated in only 3 years out of a total 12-year period of KNHANES IV–VII, but EQ-5D-3L was investigated in all 12 years. Therefore, we analyzed the risk factors for depressive symptoms, not for major depression in stroke survivors. Third, as this study was cross-sectional, the causal relationship between the risk factors and depressive symptoms could not be established. Fourth, only non-hospitalized community-dwelling stroke survivors were included, and inpatient stroke patients were excluded. Therefore, the impact of stroke on depression might have been underestimated. However, despite these limitations, the present study provides worthy information because complex sample analysis was performed using a national population-based survey.

## 5. Conclusions

Independent risk factors for depressive symptoms in stroke survivors were female sex, unemployment, low education, low family income, and activity limitations. Activity limitations were identified as the most significant risk factor, and its most common causes were stroke sequelae and musculoskeletal problems. To reduce the depressive symptoms in stroke survivors, clinicians should focus on minimizing the stroke sequelae in the acute to subacute phase and musculoskeletal problems in the chronic phase.

## Figures and Tables

**Table 1:** Demographic and clinical characteristics of the study participants.

Variable	Stroke Survivors (N = 1558)	Non-Stroke (N = 32,433)	*p*
Age (years)	67.5 ± 10.0	50.8 ± 16.7	<0.001 *
Body mass index (kg/m^2^)	24.5 ± 3.3	23.9 ± 3.5	<0.001 *
Male sex	808 (51.9%)	13,840 (42.7%)	<0.001 *
Hypertension	1064 (80.0%)	7451 (23.0%)	<0.001 *
Diabetes mellitus	451 (44.2%)	2886 (8.9%)	<0.001 *
Hyperlipidemia	489 (49.4%)	5234 (16.2%)	<0.001 *
Education level			<0.001 *
Elementary school graduate and lower	876 (56.8%)	6934 (21.5%)	
Higher than elementary school graduate	665 (43.2%)	25,296 (78.5%)	
Employment status			<0.001 *
Unemployed	1068 (69.4%)	12,823 (39.8%)	
Employed	470 (30.6%)	19,428 (60.2%)	
Marital status			<0.001 *
Unmarried	37 (2.4%)	5362 (16.5%)	
Married	1520 (97.6%)	27,069 (83.5%)	
Family income			<0.001 *
Quartile 1 (lowest)	725 (47.1%)	6092 (18.8%)	
Quartile 2	387 (25.2%)	7999 (24.7%)	
Quartile 3	243 (15.8%)	8886 (27.5%)	
Quartile 4 (highest)	183 (11.9%)	9343 (28.9%)	
Activity limitations	625 (40.4%)	2617 (8.1%)	<0.001 *
Depressive symptoms	404 (26.1%)	3369 (10.4%)	<0.001 *

Variables are presented as mean ± standard deviation or number (%). * *p* < 0.05.

**Table 2:** Comparison of the participants’ mental health according to their history of stroke.

Variable	Stroke Survivors	Non-Stroke	*p*
Patient Health Questionnaire-9	4.4 (4.0–4.8)	2.6 (2.5–2.6)	<0.001 *
Prevalence of major depression ^†^ (%)	16.2 (14.0–18.8)	5.3 (5.0–5.6)	<0.001 *
Prevalence of depressive symptoms (%)	24.7 (23.2–26.3)	9.3 (9.0–9.6)	<0.001 *
Prevalence of antidepressant treatment (%)	3.8 (3.4–4.3)	1.4 (1.3–1.5)	<0.001 *
Prevalence of suicidal ideation during the past year (%)	21.7 (20.5–23.1)	4.8 (4.4–5.1)	<0.001 *
Prevalence of a suicide attempt during the past year (%)	2.5 (2.0–3.2)	0.6 (0.5–0.6)	<0.001 *

Variables are presented as weighted means (95% confidence interval) or weighted percentages (95% confidence interval). ^†^ Major depression was defined as a PHQ-9 score of 10 or higher. * *p* < 0.05.

**Table 3:** Complex sample logistic regression analysis for depressive symptoms in stroke survivors.

Variable	Univariate Analysis		Multivariate Analysis	
	OR (95% CI)	*p*	OR (95% CI)	*p*
Age (per year)	1.012 (1.004–1.020)	0.002 *	0.995 (0.986–1.004)	0.285
Female gender	1.480 (1.257–1.743)	<0.001 *	1.381 (1.157–1.649)	<0.001 *
Body mass index (per kg/m^2^)	0.981 (0.957–1.005)	0.125	N/A	
Hypertension	0.968 (0.750–1.248)	0.800	N/A	
Diabetes mellitus	1.068 (0.862–1.323)	0.548	N/A	
Hyperlipidemia	1.151 (0.906–1.463)	0.248	N/A	
Unemployed	1.869 (1.511–2.312)	<0.001 *	1.229 (1.012–1.492)	0.037 *
Unmarried	1.283 (0.886–1.858)	0.187	N/A	
Low education level	1.682 (1.421–1.990)	<0.001 *	1.338 (1.064–1.683)	0.013 *
Family income		<0.001 *		<0.001 *
Quartile 1 (lowest)	Reference		Reference	
Quartile 2	0.697 (0.583–0.834)		0.776 (0.636–0.946)	
Quartile 3	0.473 (0.399–0.561)		0.601 (0.493–0.732)	
Quartile 4 (highest)	0.500 (0.334–0.746)		0.812 (0.541–1.219)	
Activity limitations	3.488 (2.975–4.090)	<0.001 *	3.225 (2.731–3.809)	<0.001 *

OR, odds ratio; CI, confidence interval; N/A, not applicable; Low education level, elementary school graduate and lower. * *p* < 0.05.

**Table 4:** Causes of activity limitations reported by stroke survivors (n = 625).

Causes	Number (%)
Stroke	337 (53.9)
Musculoskeletal	256 (41.0)
Vision	58 (9.3)
Hypertension	53 (8.5)
Diabetes mellitus	45 (7.2)
Aging	41 (6.6)
Mental	37 (5.9)
Heart	31 (5.0)
Oral	30 (4.8)
Respiratory	28 (4.5)
Hearing	26 (4.2)
Dementia	13 (2.1)
Others	43 (6.9)

Duplicate responses were allowed.

## Data Availability

The raw data are available at https://knhanes.kdca.go.kr/knhanes/sub03/sub03_02_05.do (accessed on 2 August 2021).
